# Assessment of Corrosion Protection Performance of FeOOH/Fe_3_O_4_/C Composite Coatings Formed In Situ on the Surface of Fe Metal in Air-Saturated 3.5 wt.% NaCl Solution

**DOI:** 10.3390/ma16010224

**Published:** 2022-12-26

**Authors:** Lina Huang, Qi Luo, Yan He

**Affiliations:** 1 Guangdong Provincial Key Laboratory of Durability for Marine Civil Engineering, Shenzhen Durability Center for Civil Engineering, College of Civil and Transportation Engineering, Shenzhen University, Shenzhen 518060, China; 2 School of Civil Engineering, Suzhou University of Science and Technology, Suzhou 215011, China

**Keywords:** carbon materials, metal composite materials, corrosion inhibition

## Abstract

The purpose of this work is to evaluate the corrosion-inhibition behavior of deposited carbon and some iron-oxide hybrid coatings which derived from the in situ deposition method on the surface of Fe foil. Various contents of precursor methane gas were deposited over a mild iron foil substrate and formed different composites. It was found that the incorporation of C into the Fe matrix led to a thin film on the surface of the matrix and produced an anti-corrosion effect. Electrochemical impedance spectroscopy (EIS), potentiodynamic polarization and potentiometric tests were used to compare the corrosion behaviors of the films in air-saturated 3.5 wt.% NaCl solution. According to the results, Fe-oxide- and C-composite-coated iron foil has a much higher corrosion resistance than the raw blank sample without the addition of C. Generally, the corrosion charge transfer resistance of one kind of iron oxide coated with carbon layers of several nanometers was enhanced up to 28,379 times (R_ct_ changes from 1487 Ω cm^2^ to 4.22 × 10^7^ Ω cm^2^), which is the biggest improvement so far. The maximum protection efficiency was obtained for the in situ grown coating prepared by 10 and 15 sccm CH_4_ precursor gas (eta = 100%). In conclusion, an iron oxide and carbon composite was found to be a great candidate for applications in the corrosion-resistance area.

## 1. Introduction

Reinforced concrete has a major problem with deterioration, which has been considered as the most important reason for concrete structure. So far, this issue remains unsolved. Although the reinforcement could be protected by stable films/coatings formed onto the surface, the passive coatings become unsteady because of chloride access and carbonation process in the marine environment, which could facilitate the corrosion procedure with the occurrence of oxygen and water [1,2,3,4,5]. The result is structural degradation happens and causes a lessened service life.

For avoiding rebar corrosion, some usually used commercial inorganic corrosion inhibitors, such as sodium nitrite, have been adopted in cathodic protection as well as stainless-steel strengthening bar (rebar). Nevertheless, these inorganic corrosion inhibitors are not friendly for the environment and are toxic, which could have a negative effect on people’s health. Researchers should focus on corrosion inhibitors which are less toxic [6,7,8]. Besides, their anti-corrosion effect is still not faultless owing to the binding of nitrite ions with the hydrated cement. However, works on the protective efficiency of green inorganic corrosion inhibitors in the application of concrete are rare. In view of this, to solve the above problem, in our work, we have prepared inorganic composites without the poison, and meanwhile they have a better effect for resistance to metal corrosion.

A substitute solution is to apply more novel, green and effective materials for achieving the anti-corrosion effect. Here, we have exploited a new method for obtaining a kind of green inorganic inhibitor which has strong anti-corrosion performance. The study has evaluated the effects of cementite on the corrosion resistance in a simulated porous solution. Based on the electrochemical values from the accelerated corrosion measurement, our work has completed an outstanding outcome for corrosion resistance during chloride attack. The work has displayed the feasibility of the adoption of cementite to inhibit steel corrosion through the adjustment of the [Cl^−^]/[OH^−^] ratio [9,10]. As a result, it is essential to apply this kind of material in cementitious materials to sweep the influence on corrosion activity and appraise the protective efficacy further toward rebar corrosion, which is the primary target for the present research. For evaluating the efficiency of the material, the most usual methods are electrochemical-based routes, such as linear sweep voltammetry (LSV), which could provide indirect electrochemical parameters indicating the instantaneous state of the corrosion reactions [11,12,13,14]. 

In this study, chemical vapor deposition equipment is adopted, which uses CH_4_ as the source of the carbon, and H_2_ is used as the reducing gas. The different contents of CH_4_ gas could obtain different forms of the composites formed onto the surface of iron foil, and lead to different effects on corrosion inhibition. The anti-corrosion mechanism of the system is to form a steady film on the surface of the rebar to protect the rebar, thus retarding the corrosion process, hence bringing about a decrease in the corrosion rate [15,16,17]. 

## 2. Experimental Procedure

### 2.1. Preparation of Coated Samples

In this study, flat iron foil with a thickness of 25 μm is used as the substrate, and the purity is 99.5%. The iron foil was purchased from Sigma-Aldrich Co., St. Louis, MO, USA. In summary, we synthesize the cementite composite layer on Fe foil by CVD in a commercial reactor. The Fe foil is annealed in H_2_ (50 sccm (mL/min)) flow for 30 min at T = 1010 °C, and different volumes of CH_4_ gas as the carbon source are inserted at 5, 10 and 15 sccm (mL/min); the corresponding samples are denoted as 1#, 2# and 3#. For comparison, the raw Fe foil is denoted as 0#.

### 2.2. Characterization

The SU8010 FESEM equipped with EDS was used to characterize the microstructure and component of the samples. In addition, HRTEM patterns are obtained by using JEM2100PLUS (JEOL Ltd., Tokyo, Japan) transmission electron microscopy. For identifying the phase composition of the coatings, XRD and Raman spectroscopy (RS) were adopted. XRD analysis was conducted by using Ultima IV equipment with Cu Kα radiation at the voltage and current of 40 kV and 40 mA. RS was carried out via LabRAM HR Evolution machine (Longjumeau, France) at 532 nm. X-ray photoelectron spectroscopy was implemented by using ESCALAB 250xi equipment. It is worthy to note that the TEM images were taken by a Talos F200s TEM operated at 200 kV. TEM sample was prepared by a dual beam FIB microscope (Strata 400S, FEI, Eindhoven, The Netherlands). Before FIB application, the top surface of the sample was coated with a 20 nm Au protection layer in a sputtering coating machine (NSC-4000, Austin, TX, USA).

### 2.3. Electrochemical Corrosion Measurements

EIS and potentiodynamic polarization tests were implemented with a Shanghai Chenhua CHI660E (Shanghai, China) in a three-electrode system. Its working electrode is coated-carbon steel, its reference electrode is Ag/AgCl and its counter electrode is platinum metal. The exposed surface area of all the tests was 1 cm^2^. To attain a stable state between the exposed sample and electrolyte, the specimens were immersed in air-saturated 3.5 wt.% NaCl solution for 12 h before each kind of test. Among them, the EIS test was carried out with a frequency range from 0.01 to 100 kHz. The polarization test was completed at a potential of 200 mV relative to the open circuit potential (OCP), and the scanning rate was 1/6 mV/s. EIS and polarization data were simulated by using Zview 3.1 and Nova software, respectively. In general, the scattering of fitted EIS data was less than 5%.

## 3. Results and Discussion

### 3.1. Characterizations of FeOOH/Fe_3_O_4_/C@Fe Composites

**Figure 1:**
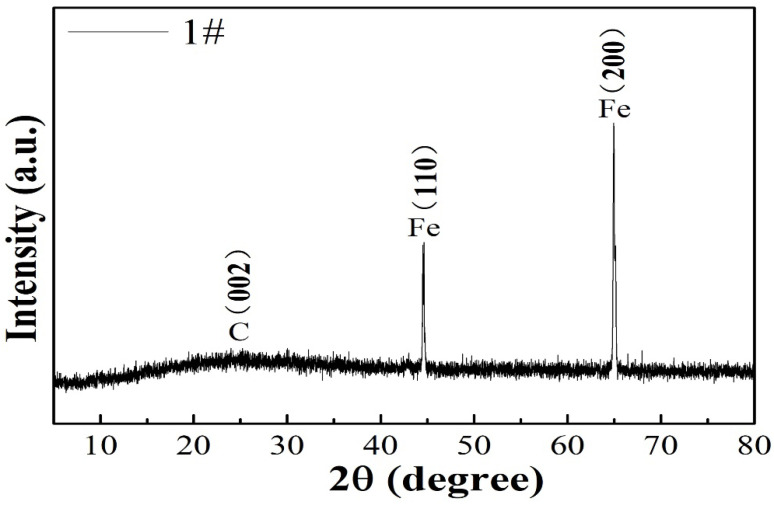
XRD pattern of 1# composite.

The structural characteristics of the 1# composite was identified by XRD. The diffraction peak at 26.3° was attributed to the (002) plane of graphitic carbon. Besides the characteristic diffraction peak of the carbon (002) plane, all the FeOOH/Fe_3_O_4_/C@Fe composites displayed two sharp peaks at about 44.79° and 65.21°, which correspond to the (110) and (200) characteristic planes of cubic Fe crystal (JCPDS card no. 06-0696) derived from the Fe foil substrate, which is shown in Figure 1. As the film thickness on the Fe foil is only several nanometers, which is beyond the detection range of XRD, it cannot be detected [18,19].

**Figure 2:**
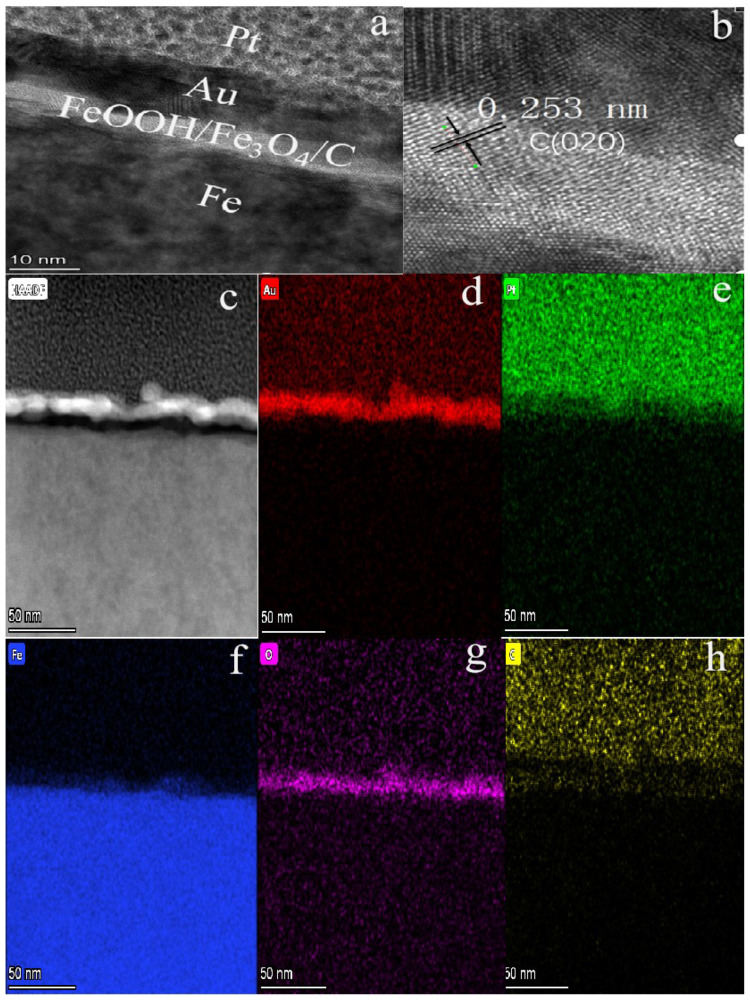
(**a**,**c**) TEM images of 1# composite; (**b**) HRTEM image of 1#; (**d**–**h**) the corresponding elemental mapping images of Au, Pt, Fe, O and C elements for 1# composite.

Figure 2a is a typical transmission electron microscopy (TEM) image of the typical FeOOH/Fe_3_O_4_/C@Fe material which displayed a thin film structure formed on pure Fe foil.

**Figure 3:**
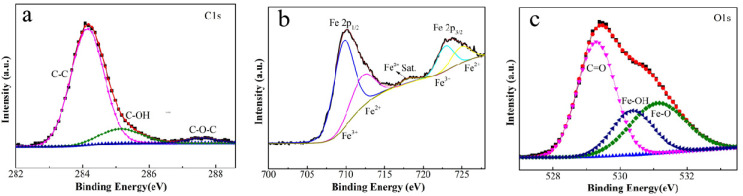
The high-resolution XPS spectra of C1s (**a**), Fe2p (**b**) and O1s (**c**) for 1# sample.

Besides these characterizations, it can be concluded that the film contents include FeOOH, Fe_3_O_4_ and some graphitic carbon by the XPS analysis of the element content on the surface of iron foil. The high-resolution spectra of C1s, Fe2p and O1s for 1# composite are presented in Figure 3. Fe, C and O elements are the main film-forming elements. The peak of Fe2p and O1s indicates the presence of FeOOH and Fe_3_O_4_. The C1s (shown in Figure 3a) can be fitted with three peaks at 284.3, 285.2 and 287.4 eV, which are assigned to the peak of C-C, C-OH and the amorphous carbon C-O-C on the coating film, respectively. The Fe2p spectrum shown in Figure 3b splits into two peaks at about 710.2 eV (Fe2p3/2) and 723.8 eV (Fe2p1/2), due to the spin orbit coupling. As displayed in the XPS spectrum of O1s (displayed in Figure 3c), the O1s could be fitted with three peaks at 529.3, 530.4 and 531.2 eV, which correspond to the peak of C=O, Fe-OH and Fe-O, respectively [20,21,22].

### 3.2. Corrosion Inhibition Capacity

**Figure 4:**
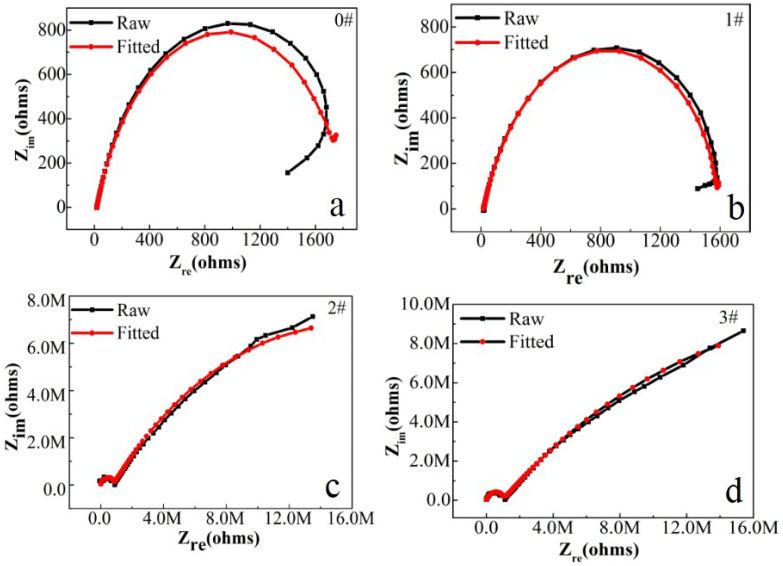
(**a**–**d**) Nyquist plots for iron foil in 3.5 wt.% NaCl aqueous solution with different contents of CH4 precursor gases: (**a**) 0#; (**b**) 1#; (**c**) 2#; (**d**) 3#.

**Figure 5:**
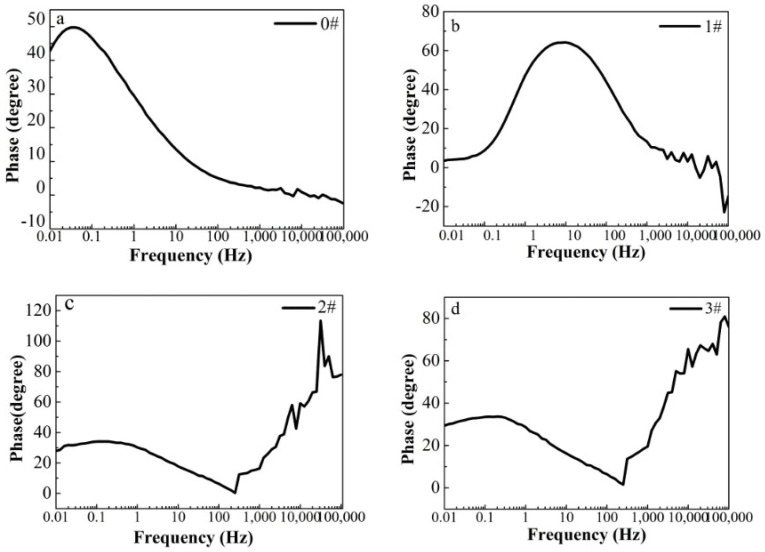
(**a**–**d**) Bode phase curves for iron foil in 3.5 wt.% NaCl aqueous solution with different contents of CH_4_ precursor gases: (**a**) 0#; (**b**) 1#; (**c**) 2#; (**d**) 3#.

**Figure 6:**
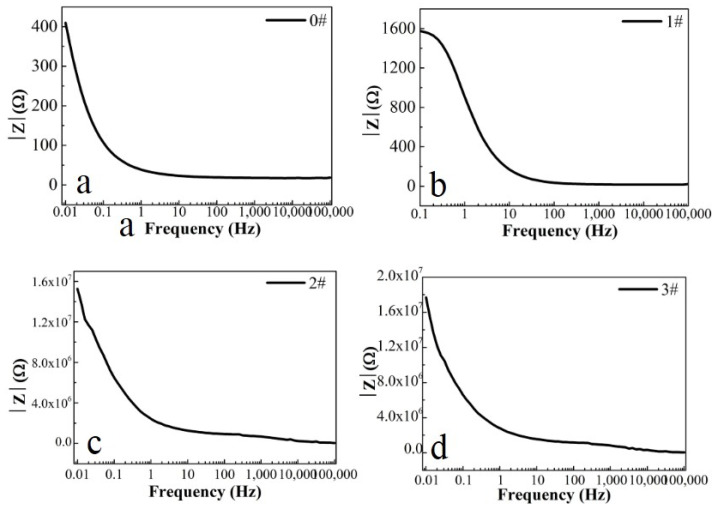
(**a**–**d**) Bode magnitude curves for iron foil in 3.5 wt.% NaCl aqueous solution with different contents of CH4 precursor gases: (**a**) 0#; (**b**) 1#; (**c**) 2#; (**d**) 3#.

In this paper, EIS measurement was performed to evaluate the corrosion-resistance performance of the composite under three fabrication conditions. Figure 4, Figure 5 and Figure 6 illustrate the Nyquist and Bode curves of different samples immersed in saline solutions containing different additives. The protective performance of the coatings can be preliminarily characterized by the impedance arc semicircle diameter in the Nyquist plot [23,24,25]. In general, higher frequencies and lower frequencies correspond to the outer and inner layers, respectively. The impedance arc of 3# coating presented larger at the low frequency, followed by 2#, while the blank sample and 1# film are nearly the same from high frequency to low frequency. It is usually identified that, in the Bode curves of impedance modulus vs. f, the low-frequency impedance value of 0.01 Hz is mainly used to evaluate the overall corrosion resistance of the coatings and the impedance modulus in the high frequency domain is usually used to characterize the properties of the measured film [26,27]. All coatings exhibited a higher value of |Z| value than Fe foil, and it indicates higher inhibition on charge transfer between the Fe substrate and the prepared film mainly due to the presence of the dense film that separates the vulnerable metal substrate from corrosive species to a large extent, especially for 3# composite that displays the highest impedance value, which can be attributed to the emergence of a steady passive coating [28]. In addition, it is interesting that, with the increased sccm, the impedance increases dramatically from 5 sccm to 10 sccm whereas it becomes relatively steady from 10 to 15 sccm. The maximum |Z|_0.01Hz_ for the 3 # sample (1.78 × 107 Ω cm^2^) indicating that the 3 # composite coating has the favorite corrosion protection among the acquired films [29,30].

For further investigation in a quantitative way, the Nyquist diagrams were fitted with appropriate equivalent electrical circuits (EECs), and their corresponding electrochemical parameters are listed in Table 1. In the EECs, Rs indicates the solution resistance, R*_f_* indicates the outer layer resistance and CPE*_f_* indicates the outer layer capacitance response. Rct represents the charge transfer resistance of the inner compact layer and CPE_dl_ represents the capacitance response of the inner compact layer. Herein, the non-ideal resistance and capacitance behavior of the coating samples is characterized by the constant phase element (CPE) as Q, due to the homogeneity of the outer and inner layers [31,32,33]. Because of the inhomogeneity of the surficial films except for their porous characteristics, the ideal capacitance (C) was replaced by a constant phase element. The CPE consists of Y0 (admittance) and n (exponent). The impedance of the CPE is calculated by (Formula (1)) [34]:

Z_CPE_ = [Y(jω)^n^]^−1^ (1)

where j^2^ = −1, ω represents the angular frequency with the maximum imaginary impedance and 0 < n < 1, which represents a non-ideal capacitor. Generally speaking, the fitted results are accepted when the error of fitting parameter χ^2^ is ≤ 5 × 10^−3^ [34].

**Figure 7:**
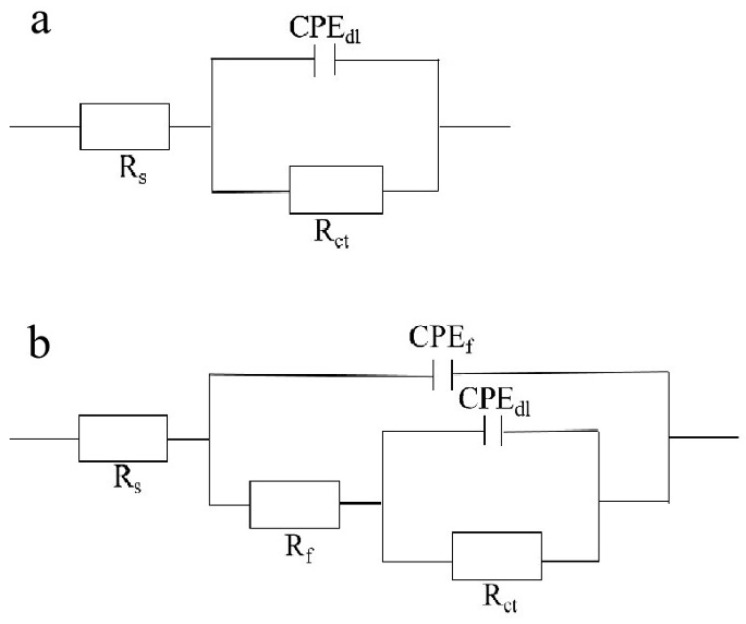
(**a**) Equivalent circuit model of EIS of blank 0# sample; (**b**) of 1#–3# samples. Among them, R_s_ denotes solution resistance; R*_f_* denotes film resistance and CPE*_f_* denotes film capacitance.

**Figure 8:**
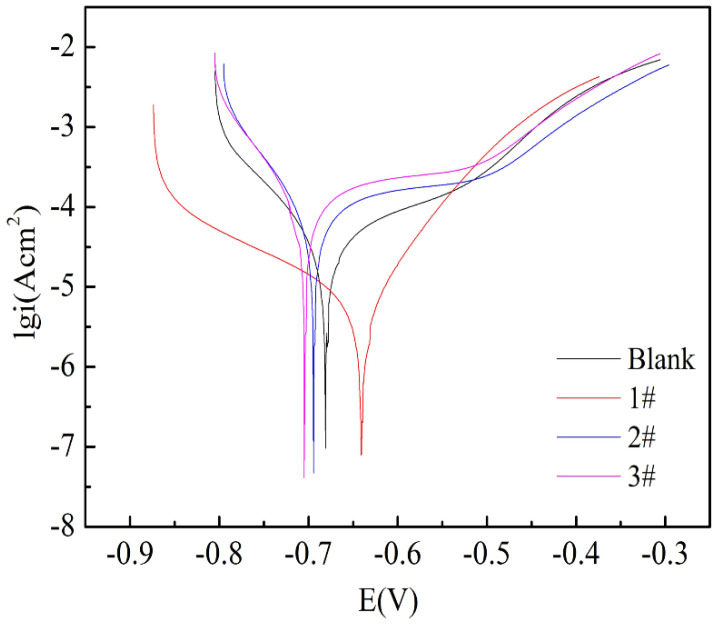
Tafel curves of blank sample and 1# to 3# composite samples.

In this table, R_ct_, charge transfer resistance, is the key parameter to assess the corrosion resistance performance of the sample [35,36,37,38,39]. Additionally, the double-layer capacitance between the coating (or the electrolyte for the blank sample) and the Fe foil is represented by CPEdl, as shown in Figure 7. The clear curves cause the perception of the 2# and 3# samples to have a second time constant at an earlier frequency, which can be used for the adsorption of nanofilm on the surface of Fe foil [40]. Inhibition of corrosion by charge transfer can be confirmed by the one-time constant curve of the blank sample [41]. According to the results, in the 3# film composite, the Rct value was around 3.37 × 10^7^ Ω cm^2^ after one hour from immersion, which is much higher than the blank sample, and it is also the maximum value in the literature so far [42,43,44]. These results show that, in the case of the 3# sample, the nano carriers had corrosive moieties from the beginning, which is the reason why Rt has the highest value. The protection efficiencies for the blank sample and the 1# to 3# samples are calculated and tabulated in Table 2 by using the equation [45,46]:

PE% = R_ct_ − R_ct_^0^/R_ct_ × 100% (2)

where R_ct_ and R_ct_^0^ are the total resistances for uncoated and coated FeOOH/Fe_3_O_4_/C composite film, respectively. It is discovered that the 2# and 3# samples show the highest PE%, reaching to almost 100%. Figure 8 demonstrates Tafel curves of the iron foil without and with FeOOH/Fe_3_O_4_/C composite under different fabrication conditions in 3.5 wt.% NaCl solution. Compared with bare Fe foil, the corrosion current (j_corr_) of the 2# and 3# samples decreased and the corrosion potential (E_corr_) shifted negatively. In addition, the other parameters (j_corr_, E_corr_, and η%) derived from the Tafel analysis results for the three samples are also displayed in Table 2. It is worth mentioning that η% for the 1#, 2# and 3# samples are 4.5, 100 and 100%, respectively. The thicker coating enhanced the corrosion-inhibition efficiency of raw iron foil, thus the reinforcement of its compactness and steadiness [47,48,49,50].

**Figure 9:**
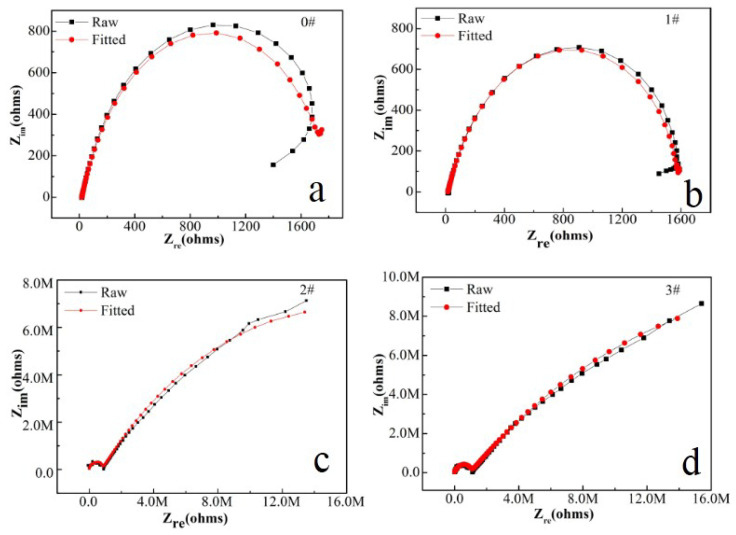
Linear polarization curves of (**a**) blank sample; (**b**) 1# sample; (**c**) 2# sample; (**d**) 3# sample of FeOOH/Fe_3_O_4_/C@Fe composites.

Except for the analysis of EIS results, the linear potentiodynamic polarization measurements are adopted to predict the corrosion resistance of the obtained coatings and the bare Fe metal, which are displayed in Figure 9. Compared with bare Fe metal, curves for all coatings moved to smaller abscissa current, which means decreased corrosion current density despite no evident changes in their corrosion potential and, by fitting the linear polarization plot within ±10 mV versus open circuit potential, the linear polarization resistance (*R*_p_) was obtained, see Table 2. The results showed that the *R*_p_ values of 2# and 3# were significantly higher than those of 1# and blank samples. It means that they have formed a protective layer on the Fe foil surface. Such a result and trend are in line with the analysis of EIS and Tafel measurement, so it is safe to assume that all films forming on Fe foils offer corrosion protection for raw Fe foil. The coatings exhibit passive behavior during the polarization process [51]. The corrosion rate is proportional to the passive current density for a polarization curve with passive behavior. The results of the potentiodynamic polarization measurements at all immersion times supply direct evidence that Fe foil is effectually protected by FeOOH/Fe_3_O_4_/C@Fe composite coatings.

### 3.3. Mechanism of Improvement of Corrosion Resistance

**Figure 10:**
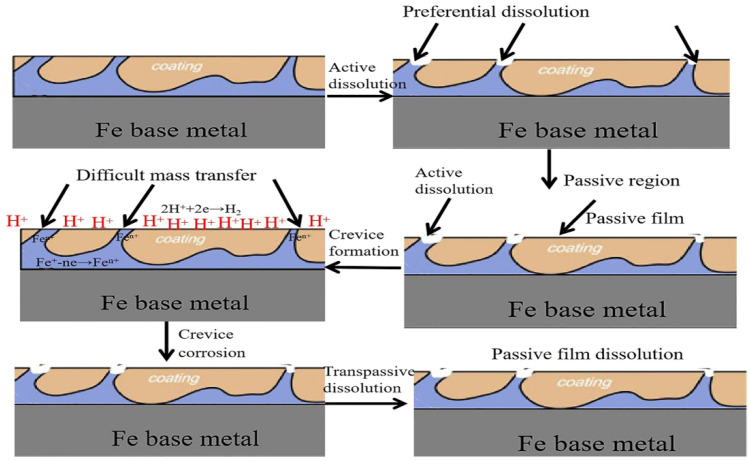
Simulated chart for the propagation of the corrosive medium through the coating during the Cl^−1^ immersion.

In order to clarify the mechanism for the enhanced corrosion resistance of the FeOOH/Fe_3_O_4_/C@Fe composite, the propagation process of the corrosive medium in the coating of FeOOH/Fe_3_O_4_/C during the immersion process is shown in Figure 9. As shown in Figure 9, the dense and uneven structure of the coatings in situ grown on the Fe base metal offers an improved barrier effect. During the immersion, chloride ions are hindered by the dense film and, merely after the surface layer is damaged, the corrosion would permeate to the interior, as shown in the final step in Figure 10.

## 4. Conclusions

In this work, a novel composite coating is designed on Fe metal by in situ grown layered different Fe oxides. The special coating separated the active Fe foil matrix from the alkaline FeOOH/Fe_3_O_4_/C composite film. The composite FeOOH/Fe_3_O_4_/C with a dense structure was formed on the Fe metal. The obtained film exhibits superb anti-corrosion performance. Moreover, the use of a carbon source, as a mixed layer on the coating surface, and the precipitation of iron as a corrosion-protective additive composite were shown to be a promising approach in real-world corrosion-security applications. 

Reasonable explanations for the improvement of corrosion resistance were considered and the following conclusions can be obtained: 1: The obtained coating on the Fe foil matrix could render a moderate corrosion barrier. 2: The maze-blocking effect of carbon coupled with the corrosion inhibition of Fe oxides will endow this system with excellent anti-corrosion performance. In conclusion, iron oxide and carbon composites were found to be a fabulous candidate for areas where high-performance films with outstanding corrosion resistance are required. 

## Figures and Tables

**Table 1:** Fitted equivalent circuit model parameters of 1#–3# samples in 3.5 wt.% NaCl aqueous solution.

Sample	R_s_ (Ω/cm^2^)	CPE-1 (S sec^n^/cm^2^)	n-1	R_coat_ (Ω/cm^2^)	CPE-2 (S sec^n^/cm^2^)	n-2	R_ct_ (Ω/cm^2^)
1#	19.8	1.03 × 10^−4^	0.8873	62.99	6.68 × 10^−5^	0.8427	1557
2#	21.7	1.09 × 10^−9^	0.7437	1.09 × 10^6^	1.56 × 10^−7^	0.4479	3.37 × 10^7^
3#	20.6	3.21 × 10^−10^	0.8335	1.01 × 10^6^	1.95 × 10^−7^	0.5101	4.22 × 10^7^
Blank	19.1	/	/	/	6.9 × 10^−5^	0.8538	1487

**Table 2:** Tafel analysis outcome for 1#−3# coatings in 3.5 wt.% NaCl aqueous solution.

Sample	E_corr_ (V)	i_corr_ (A/cm^2^)	β_c_ (mV/dec)	β_a_ (mV/dec)	I_corr_ Decrease Percentage (%)	η (%)
1#	−0.6174	2.93 × 10^−6^	−25.992	17.566	8.53	4.5
2#	−0.645	6.29 × 10^−10^	23.383	95.95	99.98	100
3#	−0.640	4.98 × 10^−10^	28.738	64.059	99.99	100
Blank	−0.616	3.18 × 10^−6^	−24.886	16.541	/	/

## Data Availability

The data presented in this study are available on request from the corresponding author.
